# Serum Vitamin A Levels in Patients with Chalazion

**Published:** 2017

**Authors:** Mohammad MALEKAHMADI, Fereydoun FARRAHI, Afshin TAJDINI

**Affiliations:** 1 Assistant Professor, Department of Ophthalmology, Jundishapur University of Medical Sciences, Ahvaz, Iran; 2 Associate Professor, Department of Ophthalmology, Jundishapur University of Medical Sciences, Ahvaz, Iran; 3 Ophthalmologist, Department of Ophthalmology, Jundishapur University of Medical Sciences, Ahvaz, Iran

**Keywords:** Serum Vitamin A, Chalazion, Recurrent Chalazion, Multiple Chalazia

## Abstract

Chalazion is a chronic, localized lipogranulomatous inflammation of the sebaceous glands of the lids. Chalazion occurs often secondary to blockage of the sebaceous gland ducts. Some studies have reported vitamin A deficiency as a risk factor for chalazion. In this study, we determined the serum levels of vitamin A in patients with chalazion. The study involved a total of 107 subjects (52 patients with chalazion and 55 control healthy subjects). The study was conducted at the Ophthalmology Clinics of Imam Khomeini Hospital, Ahwaz Jundishapur University of Medical Sciences, Ahwaz, Iran between September 2014 and February 2015. The subjects were divided into three groups according to age: 7–12 years old, 13–19 years old, and more than 19 years old. Patients were further divided into four subgroups based on the type of chalazion: single, multiple, primary, and recurrent. Blood samples were collected and the serum was tested for levels of vitamin A using high-performance liquid chromatography (HPLC). The average serum vitamin A levels in patients with chalazion in the age groups of 7–12 and 13–19 years were significantly lower than in their control counterparts. Serum vitamin A levels in patients with recurrent, multiple chalazia were significantly lower than in patients with primary, multiple chalazia (P = 0.026) and patients with a recurrent, single chalazion (P = 0.029). In conclusion, chalazion could be one of the ocular presentations of vitamin A deficiency.

## INTRODUCTION

Chalazion is a chronic, localized lipogranulomatous inflammation involving either the meibomian or Zeis glands. It occurs often secondary to non-infectious obstruction of the sebaceous gland ducts [1]. Several risk factors, including blepharitis, rosacea, gastritis, anxiety, irritable bowel syndrome, smoking, and infection by viruses or *Demodex brevis*, are associated with chalazion [2-4]. Some studies have also reported vitamin A deficiency as a risk factor for chalazion, especially in young subjects [5-7]. Vitamin A deficiency causes hyperkeratosis in the meibomian gland ducts and consequently leads to obstruction of these ducts [7]. Vitamin A is necessary for the normal growth, regeneration, differentiation, and stability of epithelial tissues, and vitamin A deficiency leads to loss of goblet cells, increased epidermal keratinization, and squamous metaplasia of the mucous membranes, including the conjunctiva [5]. Vitamin A-deficient rat presents structural abnormalities of the epithelial basement membrane and loose epithelial adhesion that result in poor healing of cornea [8]. It is estimated that about 140 million children worldwide have vitamin A deficiency, making it the second most prevalent nutritional disorder after protein-calorie malnutrition [9]. Up to half of the affected children will die within 1 year [10]. The extent of vitamin A deficiency varies across different parts of the globe, possibly affecting the development of chalazion differently. In this study, we determined the serum levels of vitamin A in patients with chalazion at the local level since there are few evidences in this subject.

## MATERIALS AND METHODS

This was a prospective case-control study that included 107 subjects (52 patients with chalazion and 55 control subjects). The study was conducted at the Ophthalmology Clinics of Imam Khomeini Hospital, Ahwaz Jundishapur University of Medical Sciences, Ahwaz, Iran between September 2014 and February 2015. This study has been approved by the Ahvaz Jundishapur University of Medical Sciences ethical committee (registration number 1310/D). Written consent was obtained from the parents of the child participants and from the those adult participants before blood sampling. To minimize bias and for the best age, socioeconomic, and nutritional status matching, the control subjects were selected from among the patients’ family members with the closest age difference. All subjects were divided into three groups: 7–12 years old, 13–19 years old, and more than 19 years old. Patients were further divided into subgroups based on the type of chalazion: single, multiple, primary, and recurrent. Exclusion criteria were as follows: patients with fat malabsorption diseases, chronic alcoholism, long-term zinc therapy, long-term antacid therapy, chronic laxative abuse, antihyperlipidemic drug use, colchicine therapy, and those who refused to participate in the study. Blood samples were collected and the serum was tested for levels of vitamin A using high-performance liquid chromatography (HPLC). The following concentrations were regarded as the normal ranges of serum vitamin A levels: 0.2–0.4 g/ml for 1–6 years of age, 0.26–0.4 g/ml for 7–12 years of age, 0.24–0.72 g/ml for 13–19 years of age, and 0.3–0.8 g/ml for >19 years of age. Statistical analysis was performed using the generalized linear model. For multiple comparisons, data were adjusted using a sequential Sidak correction. 

## RESULTS

The characteristics of all 52 patients with chalazion 55 control subjects presented in Table 1. There were no statistically significant differences in demographic data between patients with chalazion and control subjects (P > 0.05).

The average serum vitamin A levels in patients with chalazion and control subjects were 0.2729 ± 0.102 g/ml and 0.3129 ± 0.06751 g/ml, respectively (P = 0.0168). To minimize bias, we compared the average serum vitamin A levels in patients of each age group with the levels of the corresponding control groups. Average serum vitamin A levels in patients with chalazion were significantly lower than in their control counterparts in the age groups of 7–12 years old (0.179 ± 0.051 g/ml vs. 0.315 ± 0.069 g/ml, P = 0.002) and 13–19 years old (0.23 ± 0.079 g/ml vs. 0.35 ± 0.077 g/ml, p 0.037). There was no significant difference in the serum vitamin A levels between patients with chalazion > 19 years old and their control counterparts (0.3125 ± 0.099 g/ml vs. 0.3064 ± 0.065 g/ml, P = 0.910) (Table 2).

Thirty-three (63.5%) patients had single chalazion and 19 patients (36.5%) had multiple chalazia. Primary chalazion was seen in 33 (63.5%) patients and 19 (36.5%) patients had recurrent chalazion. Serum vitamin A levels in patients with recurrent, multiple chalazia (0.2371 ± 0.0862 g/ml) were significantly lower than those in patients with primary, multiple chalazia (0.3013 ± 0.1325 g/ml, P = 0.026) and patients with recurrent, single chalazion (0.2886 ± 0.12655 g/ml, P = 0.029).

**Table 1:** Demographic Data of Patients with Chalazion and Control Subjects

Characteristic	Patients	Controls
Age (years)		
** 7–12**	8 (15.4%)	10 (18.2%)
** 13–19**	12 (23.1%)	6 (10.9%)
** > 19**	32 (61.5%)	39 (70.9%)
Sex		
** Male**	22 (42.3%)	28 (50.9%)
** Female**	30 (57.7%)	27 (49.1%)
All	52 (100%)	55 (100%)

**Table 2:** Comparison of Serum Vitamin A Levels by Age. Data are the Mean ± Standard Deviation.

Age (years)	Serum vitamin A (g/ml)	P-value
7–12		0.002
** Patients**	0.179 ± 0.051	
** Controls**	0.315 ± 0.069	
13–19		0.037
** Patients**	0.23 ± 0.079	
** Controls**	0.35 ± 0.077	
>19		0.910
** Patients**	0.3125 ± 0.099	
** Controls**	0.3064 ± 0.065	

## DISCUSSION

Serum vitamin A levels in subjects aged 7–12 and 13–19 years old were significantly lower in patients with chalazion than in control subjects. However, there were no significant differences between the two groups in the age group of more than 19 years old. These findings suggest that low vitamin A levels play a role in the pathogenesis of chalazion in younger ages. Furthermore, the findings of this study suggest that low serum vitamin A is associated with recurrent, multiple chalazia, but not with other types. Vitamin A deficiency causes keratinization of the epithelial cells of the meibomian gland ducts, which could lead to obstruction of the ducts and accumulation of gland secretions [7]. This may be worsened due to an inflammatory process in the meibomian glands [11]. In young ages, the meibomian gland ducts are thinner and more susceptible to obstruction due to keratinization. Lower vitamin A levels are correlated with keratinization [6] and perhaps more serious obstruction, which could explain the low levels of serum vitamin A in patients with recurrent, multiple chalazia. 

In a study conducted on children from the southwest region of China, the average serum vitamin A levels of patients with single chalazion or multiple chalazia were significantly lower than those of the control group [6]. Our study also revealed low serum vitamin A levels in children with chalazion. However, in our study, recurrent, multiple chalazia, but not multiple chalazia alone, were correlated with low serum vitamin A levels. Therefore, multiple chalazia and recurrence alone do not appear to have a significant relationship with serum vitamin A levels. 

The current study has certain limitations. First, the patients were selected from single tertiary center which may lead to selection bias. Second, the sample size was small, which may lead to decreased statistical power. Therefore studies with more cases are needed to support the data from this study. 

In conclusion, serum vitamin A levels in younger ages were significantly lower in patients with chalazion than in control subjects. This information could be helpful for the early diagnosis and treatment of vitamin A deficiency, which could prevent further complications such as xerosis and nyctalopia.
